# Investigation of hypoxia networks in ovarian cancer via bioinformatics analysis

**DOI:** 10.1186/s13048-018-0388-x

**Published:** 2018-02-26

**Authors:** Ke Zhang, Xiangjun Kong, Guangde Feng, Wei Xiang, Long Chen, Fang Yang, Chunyu Cao, Yifei Ding, Hang Chen, Mingxing Chu, Pingqing Wang, Baoyun Zhang

**Affiliations:** 1 Bioengineering Institute of Chongqing University, Chongqing, China; 2 State Key Laboratory of Quality Research in Chinese Medicine, Institute of Chinese Medical Sciences, University of Macau, Macao, China; 3 Sichuan TQLS Animal Husbandry Science and Technology Co., Ltd, Mianyang, China; 4 Key Laboratory of Farm Animal Genetic Resources and Germplasm Innovation of Ministry of Agriculture, Institute of Animal Science, Chinese Academy of Agricultural Sciences, Beijing, China

**Keywords:** Ovarian cancer, Bioinformatics analyses, ErbB signaling pathway, Molecular mechanism, Hypoxia

## Abstract

**Background:**

Ovarian cancer is a leading cause of the death from gynecologic malignancies. Hypoxia is closely related to the malignant growth of cells. However, the molecular mechanism of hypoxia-regulated ovarian cancer cells remains unclear. Thus, this study was conducted to identify the key genes and pathways implicated in the regulation of hypoxia by bioinformatics analysis.

**Methods:**

Using the datasets of GSE53012 downloaded from the Gene Expression Omnibus (GEO), the differentially expressed genes (DEGs) were screened by comparing the RNA expression from cycling hypoxia group, chronic hypoxia group, and control group. Subsequently, cluster analysis was performed followed by the construction of the protein-protein interaction (PPI) network of the overlapping DEGs between the cycling hypoxia and chronic hypoxia using ClusterONE. In addition, gene ontology (GO) functional and pathway enrichment analyses of the DEGs in the most remarkable module were performed using Database for Annotation, Visualization and Integrated Discovery (DAVID) software. Ultimately, the signaling pathways associated with hypoxia were verified by RT-PCR, WB, and MTT assays.

**Results:**

A total of 931 overlapping DEGs were identified. Nine hub genes and seven node genes were screened by analyzing the PPI and pathway integration networks, including ESR1, MMP2, ErbB2, MYC, VIM, CYBB, EDN1, SERPINE1, and PDK. Additionally, 11 key pathways closely associated with hypoxia were identified, including focal adhesion, ErbB signaling, and proteoglycans in cancer, among which the ErbB signaling pathway was verified by RT-PCR, WB, and MTT assays. Furthermore, functional enrichment analysis revealed that these genes were mainly involved in the proliferation of ovarian cancer cells, such as regulation of cell proliferation, cell adhesion, positive regulation of cell migration, focal adhesion, and extracellular matrix binding.

**Conclusion:**

The results show that hypoxia can promote the proliferation of ovarian cancer cells by affecting the invasion and adhesion functions through the dysregulation of ErbB signaling, which may be governed by the *HIF-1α-TGFA-EGFR-ErbB2-MYC* axis. These findings will contribute to the identification of new targets for the diagnosis and treatment of ovarian cancer.

**Electronic supplementary material:**

The online version of this article (10.1186/s13048-018-0388-x) contains supplementary material, which is available to authorized users.

## Background

Ovarian cancer, the most lethal gynecological malignancy, is a major cause of cancer-related mortality in women, with an estimated 22,280 new cases and 14,240 deaths predicted for 2016 in the United States [1]. Ovarian cancer is often called the “silent killer” because its signs and symptoms are frequently absent until it has reached advanced stages where outcome is poor [2]. Accumulating evidence suggests that a hypoxic environment in vivo, i.e., the absence of a sufficient oxygen supply, is tightly associated with a poor prognosis and a high mortality in patients with ovarian cancer [3]. However, the detailed mechanisms by which hypoxia regulates the status of ovarian cancer cells leading to a series of physiological changes are still unknown. Therefore, exploring the effect of hypoxia on ovarian cancer cells will likely have important implications and offer opportunities to solve this problem for potential therapeutic purposes.

The oxygen tension of normal tissues is in the region of 1-4%, while hypoxia is < 1% [4]. Generally, rapidly growing tumors outstrip their vascular supply and become hypoxic. Under hypoxic conditions, tumor cells adapt by generating energy in oxygen independent ways and minimize cellular damage by inducing the expression of genes involved in angiogenesis, glycolysis, cell survival, invasion, tumor progression, and pH regulation, which can observably influence cell metabolism by activating the hypoxia inducible factor-1 (HIF-1) signaling pathway [5]. In recent decades, more and more researchers have devoted themselves to exploring the potential mechanisms by which hypoxia regulates the progression of ovarian cancer cells. For instance, it has been demonstrated that the endothelin-1/ endothelin A receptor (ET-1/ETAR) axis in epithelial ovarian cancer (EOC) cells induces vascular-endothelial growth factor (*VEGF*) expression through HIF-1α nuclear accumulation, resulting in the invasion of cancer cells [6]. Rab25, a small GTPase of the Rab11 subfamily, has been functionally linked to the progression and aggressiveness of ovarian cancer. Enhanced Rab25 expression in ovarian cancer cell lines results in increased cell proliferation, inhibition of apoptosis and anoikis, as well as increased aggressiveness in vivo [7], which has been found to rely on the activation of HIF-1 [8]. In addition, hypoxia is well-known in increasing the resistance to chemotherapy and radiotherapy and result in the decline of cell immunity, which both contribute to the survival and growth of cancer cells [9–11]. Moreover, hypoxia was found to induce the expression of *HIF-1α* and G-protein estrogen receptor (*GPER*) that were involved in the regulation of *VEGF* expression in breast cancer cells and carcinoma-associated fibroblasts (CAFs), leading to the release of angiogenic factors and the growth of new blood vessels [12]. Hypoxia can also regulate the frequency of tumor-initiating cells by promoting epithelial-mesenchymal transition (EMT) and metastasis formation [13, 14]. Overall, the effects of hypoxia on cells are regulated in a variety of ways depending on the external environment and cell type. Therefore, it is of great practical significance to explore the specific action modes and pathways of hypoxia on ovarian cancer cells.

Although ovarian cancer has been long studied from the perspective of single genes and their specific properties, it has become clear that more integrative, systematic analyses are necessary to better understand how this serious disease develops and how it may respond to hypoxia. Recently, gene expression profile data associated with ovarian cancer have been studied by many researchers. For example, Fu et al. indicated that ovarian cancer was closely associated with dysregulation of the p53 signaling pathway, drug metabolism, tyrosine metabolism, and cell cycle by screening the differentially expressed genes (DEGs) between human ovarian cancer samples and healthy controls based on the microarray data of GSE14407. Further, a series of genes, such as cyclin E1 (*CCNE1*), cyclin B2 (*CCNB2*), cytochrome P450 family 3 subfamily A member 5 (*CYP3A5*), and vascular endothelial growth factor A (*VEGFA*), have been predicted as target genes for diagnosing ovarian cancer [15]. Additionally, Xue et al. explored the molecular mechanisms of NSC319726, a newly discovered anticancer small-molecule drug, in ovarian cancer by bioinformatics analyses and found that it might play a role against ovarian cancer via targeting genes involved in the oocyte meiosis pathway, such as ribosomal protein S6 kinase A6 (*RPS6KA6*), B-cell CLL/lymphoma 6 (*BCL6*), forkhead box O3 (*FOXO3*), cyclin B1 (*CCNB1*), and cell division cycle 20 (*CDC20*) [16, 17]. However, studies have not been yet performed on the relationship between target genes and hypoxia, nor on the main effect of hypoxia on the function and relevant regulatory pathways of ovarian cancer cells. In this context, investigating the regulation of hypoxia on the progression of ovarian cancer by bioinformatics analyses supposes to be necessary.

In the present study, RNA expression in the ovarian cancer cell line SK-OV-3 were compared between untreated and hypoxia treated (including cycling hypoxia and chronic hypoxia) samples to identify the DEGs related to hypoxia effects based on the microarray datasets. Subsequently, the protein-protein interaction (PPI) network of the overlapping DEGs was constructed and the hub genes in the network with wide influence on others were identified. Then, the most remarkable module was screened followed by cluster analysis of the PPI network. Afterwards, the Gene Ontology (GO) functional and Kyoto Encyclopedia of Genes and Genomes (KEGG) (http://www.genome.jp/kegg/) pathway enrichment analyses of the most remarkable module were performed. Finally, an integrated pathway network associated with hypoxia in ovarian cancer was constructed and verified. The findings of this study improve the understanding of the role of hypoxia and initially validate a subset of these markers in ovarian cancer cells. This investigation also provides a resource for building new hypotheses for further follow-up studies.

## Methods

### Data normalization and identification of DEGs

The microarray expression profile datasets (GEO access number: GSE53012), contributed by Olbryt et al. [18], were downloaded from the National Center of Biotechnology Information (NCBI) Gene Expression Omnibus (GEO) database (http://www.ncbi.nlm.nih.gov/geo/), which was currently the largest fully public gene expression resource [19]. There were a total of nine samples in this analysis, including three untreated samples, three samples treated with cycling hypoxia and three with chronic hypoxia. After background correction and quartile data normalization were performed [20, 21], the DEGs were identified between untreated samples and cycling hypoxia treated samples (DEGs1) and between untreated samples and chronic hypoxia treated samples (DEGs2) [22], with the criteria of |log fold change (FC)| > 1 and *p*-value < 0.05 followed by multiple-testing correction using the Benjamini-Hochberg procedure to obtain the adjusted p-value [18]. Based on these data, the overlapping DEGs between DEGs1 and DEGs2 were further analyzed.

### PPI network construction

Considering that proteins rarely work alone, it is necessary to study the interactions among proteins. Therefore, the protein-protein interaction network (PPI) of the overlapping DEGs was constructed with a confidence score of > 0.4 based on the Search Tool for the Retrieval of Interacting Genes (STRING) database [23] (STRING, version 9.1, http://string91.embl.de/), which was a precomputed global resource to predicted and known interaction information, by the use of Cytoscape [24] (version 3.0; http://cytoscape.org/), which was a general bioinformatics package. In the PPI network, each node stands for a gene and edges represent the interactions between the nodes. In view of the fact that most of the networks were scale-free, the hub genes were then selected with a connectivity degree ≥35 after calculating the degree of each node.

### Module analysis of the PPI network

Module analysis of PPI network was performed with the parameters of minimum size > 5 and minimum density < 0.05 using ClusterONE, a Cytoscape plugin that unified different clustering techniques and displayed them in a single interface [25]. Subsequently, to assess the function of the overlapping DEGs at the molecular level, on the basis of the Database for Annotation, Visualization and Integrated Discovery (DAVID) [26], the genes obtained from the most significant module were selected for pathway enrichment analysis using the KEGG database, to classify the correlated gene sets into their respective pathways [27], and for functional enrichment analysis using the Gene Ontology database (GO, http://www.geneontology.org/), to collect functions of genes and gene products from the aspects of biological process (BP), molecular function (MF) and cellular component (CC) [28].

### Pathway integration

To further interpret and visualize the molecular changes of ovarian cancer in hypoxia at the pathway level, a comprehensive network that integrated the HIF-1 signaling pathway and other signaling pathways was described by applying the combined and visualized functionality in Cytoscape based on the pathway analysis of the most significant module. Furthermore, to elucidate the regulation network of hypoxia in ovarian cancer in detail, the significant pathways inextricably linked with HIF-1 signaling pathway were screened with a carrying degree larger than 3 for the further analysis.

### Gene integration

As above, a network of integrated genes from the critical pathways and the HIF-1 signaling pathway was constructed to obtain better insight into how biological processes were regulated in human ovarian cancer, which might contribute to identifying new markers and drug targets for the diagnosis and treatment of ovarian cancer. Subsequently, in order to verify the reliability of the results of the integrated network constructed by bioinformatics analysis, a signaling pathway (ErbB signaling pathway) was selected for validation through testing the mRNA expression levels of the relevant critical genes, such as transforming growth factor alpha (*TGFA*), erb-b2 receptor tyrosine kinase 2 (*ErbB2*), and v-myc avian myelocytomatosis viral oncogene homolog (*MYC*), through regulating the *HIF-1α* expression in order to establish the relationship between the HIF-1 and ErbB signaling pathways.

### Cell culture and transfection

The human ovarian cancer cell line, Caov3, a kind gift from department of pathology, cancer hospital of Guizhou province, was cultured in a monolayer in Dulbecco’s modified Eagle’s medium (DMEM) (Gibco, Life Technologies, Carlsbad, CA, USA) supplemented with 10% fetal bovine serum (FBS) and 100 mg/mL penicillin/streptomycin (Life Technologies), and was maintained at 37 °C in an atmosphere of 5% CO_2_, as previously described [29]. Then, the cells were split into 12-well plates at a final concentration of 5 × 10^5^ viable cells/ml culture medium. The medium was replaced with RPMI only after 12 h, then the overexpression vector PMX-Flag-hif-1α and interference vectors pSUPER-TGFA/EGFR were transfected using Lipofectamine 2000. 8 h later, cells were subsequently treated with the complete medium for 24 h, followed by extraction of total RNA.

### Quantitative real-time PCR

Total RNA was extracted using a TRIzol reagent kit (TaKaRa) according to the manufacturer’s protocol as described previously [30]. Subsequently, complementary DNA was synthesized from the RNA by reverse transcription using the SuperScript III First-Standard Synthesis System for Reverse Transcription-Polymerase Chain Reaction (RT-PCR) (Invitrogen Co.). Following the protocol provided by the manufacturer, the expression of the selected genes was quantified by the PCR System 7500 (Promega) and SYBR green using the following procedures: initial denaturation and enzyme activation at 95 °C for 30 s, followed by 40 cycles of denaturation at 95 °C for 5 s and annealing at 60 °C for 30 s [31]. Moreover, the melting curve was analyzed for each gene at the end of PCR. Finally, mRNA expression was normalized to β-actin. Information on the primers is provided in Table 1.

**Table 1:** The sequences of primers for quantitative RT-PCR

Genes	Primers	Length of target fragment, bp
β-actin	F: 5′-ACTCCTATGTGGGTGACGAGG-3′	137
	R: 5′-CACACGCAGCTCATTGTAGAAG-3′	
TGFA	F:5′-CAGCAGTGGTGTCCCATTTT-3′	105
	R:5′-ACCAACGTACCCAGAATGGC-3′	
EGFR	F:5′-CCGCAAAGTGTGTAACGGAA-3′	152
	R:5′-CCTGTGGATCCAGAGGAGGAG-3′	
ErbB2	F:5′-CTGCAGCTTCGAAGCCTCAC-3′	106
	R:5′-GAGAGCCAGCTGGTTGTTCT-3′	
MYC	F:5′- GGGTAGTGGAAAACCAGCAGC-3′	119
	R:5′-CTGCTGCTGCTGGTAGAAGTT-3′	

*F* Forward, *R* reverse

### Protein extraction and western blot analysis

Caov3 cells were grown to 80% confluence in six-well plates and treated with different vector combinations. After 24 h, cells were washed with cold phosphate buffered saline (PBS, Lonza), and then harvested in Radio-Immunoprecipitation Assay (RIPA) lysis buffer supplemented with protease inhibitor (Nacalai USA). Subsequently, the soluble fraction of the lysate was isolated after centrifugation (12,000 rpm for 15 min at 4 °C). Total proteins were quantified using the bicinchoninic acid (BCA) protein assay kit and separated by 10% sodium dodecyl sulphate-polyacrylamide gel electrophoresis (SDS-PAGE) according to the manufacturer’s protocol (Thermo Scientific). The proteins were then transferred onto polyvinylidene difluoride (PVDF) membranes (Bio-Rad) and blocked in 5% nonfat dry milk for 1 h. Subsequently, the membranes were incubated with the primary antibodies (rabbit polyclonal anti-GAPDH, TGFA, EGFR, ERbB2, MYC antibodies (Cell Signaling Technologies, 1:1000)) overnight at 4 °C. After three washes in TBST buffer (pH 7.6), membranes were incubated with a IgG secondary antibody (Santa Cruz Biotechnology, 1:10000) at 37 °C for 1 h and then washed three times in TBST. The immune complexes were visualized with an ECL kit (GE Healthcare) after exposure to a Biomax film (ISC Biosciences).

### Cell proliferation assay

To evaluate the effects of these critical genes and signaling pathways on growth of ovarian cancer cells, 3-(4,5-dimethyl-2-thiazolyl)-2,5-diphenyl-2-H-tetrazolium bromide (MTT) assays were performed to assess and compare the multiplication capacities of the different treatment groups. Cells were counted and seeded in 96-well plates in triplicate for 12 h. 20 ul of MTT solution was added to each well with a final concentration of 5 mg/ml, before an additional incubation for 4 h at 37 °C. Thereafter the medium was removed and 200 ul DMSO was added to each well in order to dissolve the dye. After the formazan crystals had dissolved, the absorbance was determined at 570 nm by Thermo Scientific Varioskan Flash (Thermo, USA).

### Statistical analysis

All values are given as mean ± SEM of at least three independent experiments. Statistical significance was assessed by one-way ANOVA followed by Tukey’s multiple comparisons test using Prism statistical software (GraphPad Software, Inc., La Jolla, CA), with differences considered significant at the level of *P* < .05.

## Results

### Identification of the DEGs

**Fig. 1:**
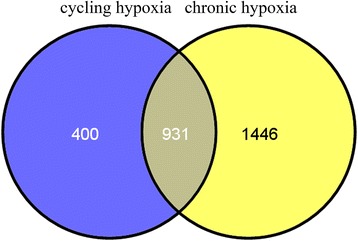
Schematic venn diagram of differentially expressed genes

After data preprocessing, a total of 1331 DEGs were identified in the samples treated with cycling hypoxia compared to untreated cells; of these, 791 were up-regulated and 540 were down-regulated. Furthermore, 2377 DEGs were screened by comparing chronic hypoxia with normal conditions, in which 1280 genes were up-regulated. According to the above data analysis, 931 overlapping DEGs were identified between cycling hypoxia and chronic hypoxia (Fig. 1).

### PPI network construction and hub gene identification

**Fig. 2:**
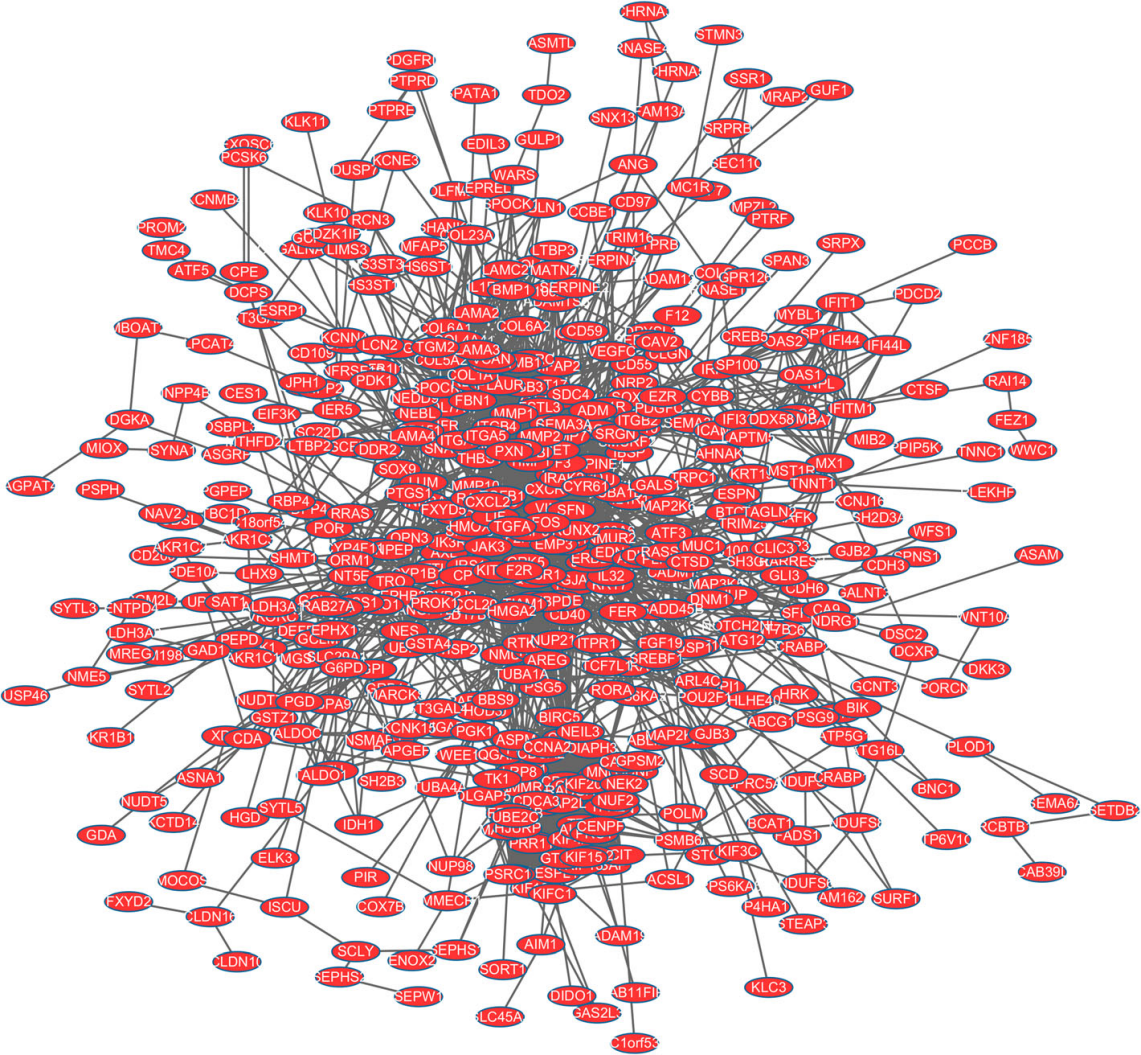
The PPI network of overlapping DEGs under cycling hypoxia and chronic hypoxia in ovarian cancer. Nodes stand for the proteins (genes), and edges stand for the interactions of proteins

On the basis of the information obtained from the STRING database, the PPI framework containing a total of 3279 protein pairs and 603 nodes were generated with the threshold of combined score > 0.4 (Fig. 2), in which nodes represented proteins and edges represented interactions between proteins [32]; this is helpful for understanding the regulation of hypoxia in ovarian cancer in the aspect of proteomics. Furthermore, proteins with very high degree may play a central regulatory role in this process, and are commonly called “hubs”. Therefore, the hub genes of the PPI network were screened with the cut off criterion of connectivity degree ≥35. The results of some nodes are shown in the Table 2, including jun proto-oncogene (*JUN*), FBJ osteosarcoma oncogene (*FOS*), estrogen receptor 1 (*ESR1*), matrix metallopeptidase 2 (*MMP2*), erb-b2 receptor tyrosine kinase 2 (*ErbB2*), and v-myc avian myelocytomatosis viral oncogene homolog (*MYC*).

**Table 2:** The statistical results of connectivity degrees of the PPI network

Gene	Degree
JUN	69
FOS	69
BIRC5	63
ESR1	59
MMP2	50
ERBB2	47
E2F7	41
MYC	37
VIM	37

The gene in the table is the symbol of the protein (gene). Degree stands for the connectivity degree of the gene

### Module analysis of the PPI network

**Fig. 3:**
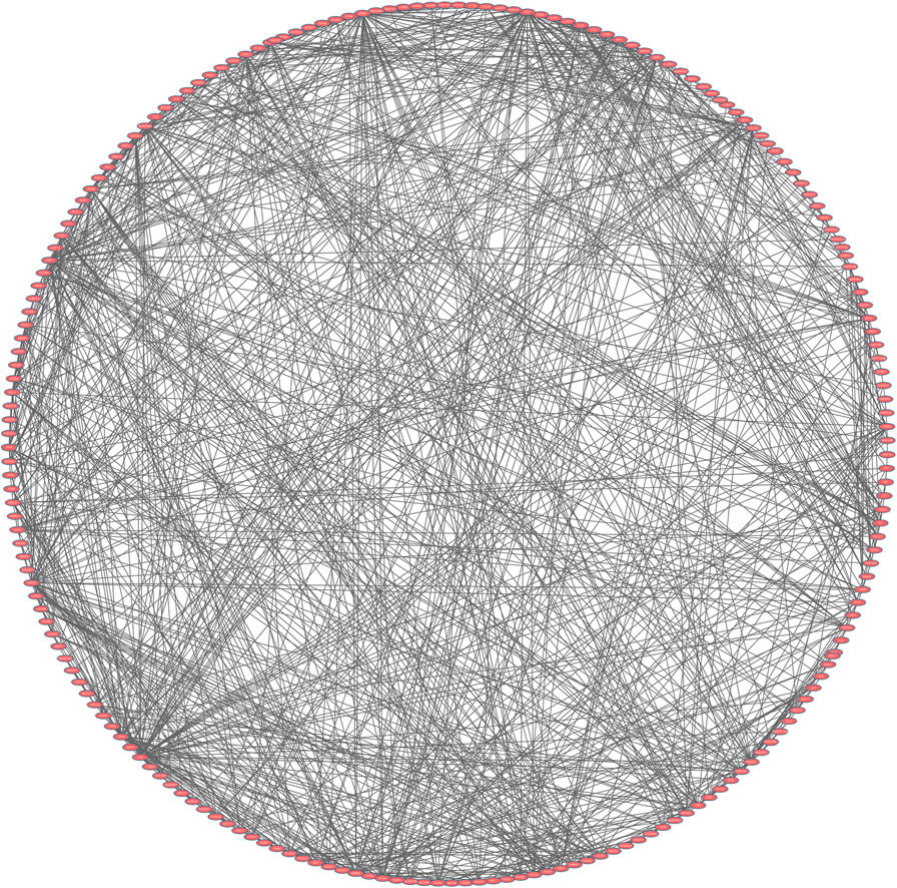
The most significant module in the PPI network. Nodes stand for the proteins (genes), and edges stand for the interactions of proteins

**Fig. 4:**
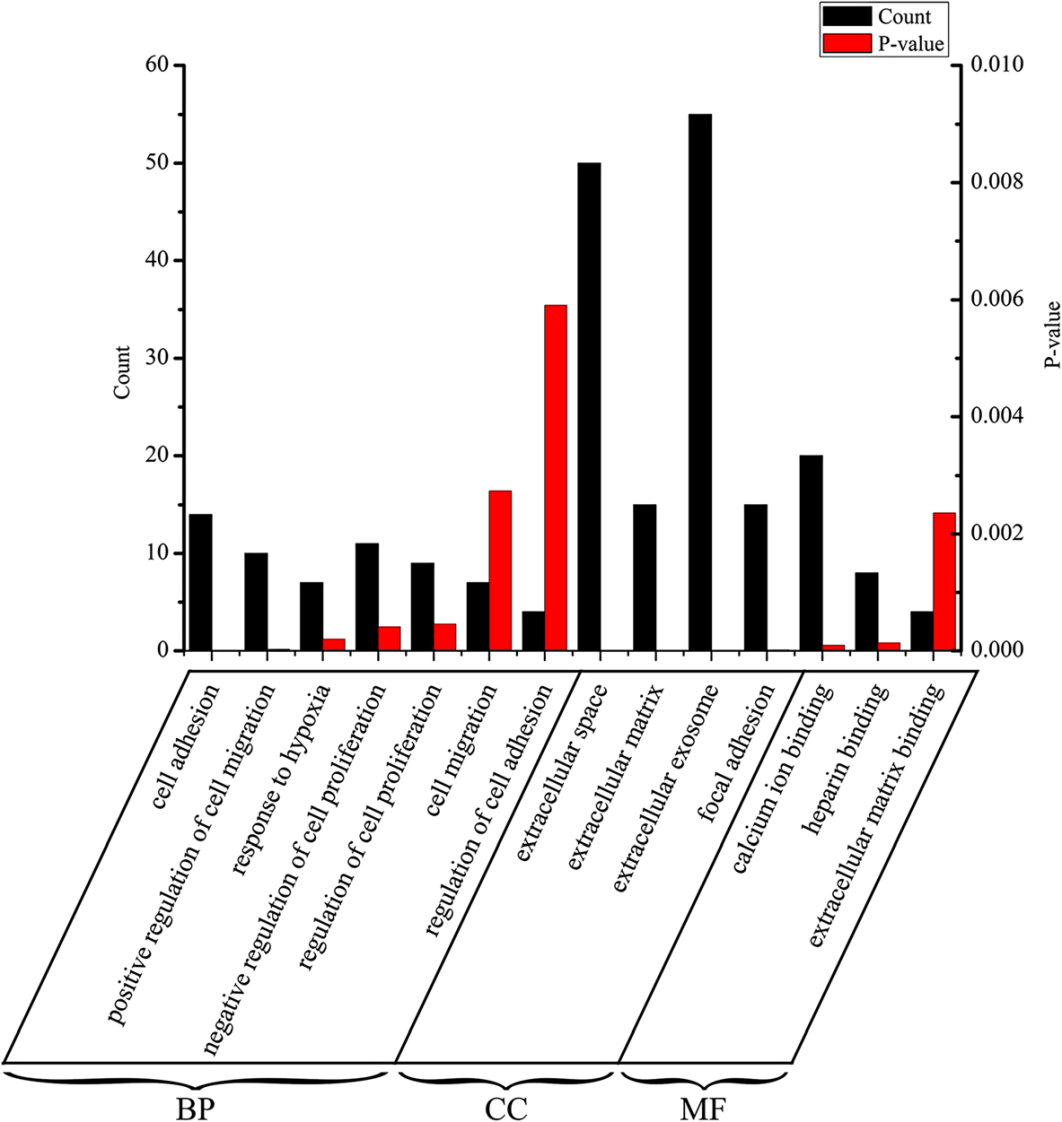
The GO analysis of the most significant module. The left ordinate of histogram represents the gene counts, and the right represents the *P*-value. BP stands for biological process; MF stands for molecule function; and CC stands for cellular component

To study and identify the function of the overlapping DEGs in detail, cluster analysis of the PPI network was conducted based on the ClusterONE Cytoscape plugin, an important tool for the analysis of densely connected and possibly overlapping regions within the Cytoscape network, which would contribute to the classification of protein network and relevant analysis. There were a total of 51 functional modules given with the parameters of minimum size > 5 and minimum density < 0.05 and the most significant module (nodes = 223, density = 0.051, quality = 0.802, *p* value = 0.000, Fig. 3) was selected for the further analysis of functions and pathways to deeply understand the main effect of hypoxia in the course of ovarian cancer progression. Based on DAVID, GO functional annotation of genes obtained from the most significant module was performed (Fig. 4) and found that they mainly participated in cell proliferation, migration, and adhesion. These results suggest hypoxia may promote the proliferation of ovarian cancer cells by affecting cell invasion and adhesion. To further verify the accuracy of this inference, the module genes were submitted into DAVID to perform the KEGG pathway enrichment analysis. The results showed that they were significantly enriched in PI3K-Akt, MAPK, Wnt and ErbB signaling pathways, as well as ECM-receptor interactions, focal adhesion, pathways in cancer. Not surprisingly, the HIF-1 signaling pathway was also enriched (Additional file 1: Figure S1). It is well established that these signaling pathways were closely related to cell proliferation, differentiation, and apoptosis. The proliferation and clone-forming ability of periodontal ligament stem cells (PDLSCs) were markedly enhanced by hypoxia, which may be implicated in the activation of p38/MAPK and ERK/MAPK signaling pathways [33]. In addition, the PI3K-Akt and Wnt signaling pathways have been reported to play an important role in the process of mesenchymal stem cells (MSCs) and MC3T3-E1 cell proliferation induced by hypoxia [34–37]. It is noteworthy that hypoxia-induced cell proliferation was also mediated by increased fibronectin (*FN*), integrin β1 (*IN β1*) as well as extracellular matrix (E*CM*) expression through the PI3K/Akt, mTOR, and HIF-1 pathways, followed by focal adhesion kinase (FAK) activation [38, 39]. Moreover, hypoxia can inhibit anoikis by maintaining ErbB signaling pathway to promote cell proliferation [40]. Taken together, these results further indicate that hypoxia has an influence on the proliferation of cells through multiple signaling pathways, which may be associated with the changes of the function of invasion and adhesion.

### Pathways integration

The physiological function of organism is the result of the coordination of many kinds of signaling pathways. Therefore, it is critical to identify genes and their protein products that share common pathways. Thus, the HIF-1 signaling pathway and other signaling pathways were integrated by the use of Cytoscape (Additional file 2: Figure S2). Then, to provide insights into the effect of hypoxia on ovarian cancer cell proliferation, the genes linked to these signaling pathways were identified, including *ErbB2*, phosphatidylinositol-4,5-bisphosphate 3-kinase catalytic subunit delta (*PIK3CD*), phosphoinositide-3-kinase regulatory subunit 3 (*PIK3R3*), endothelin 1 (*EDN1*), cytochrome b-245 beta chain (*CYBB*), serpin family E member 1 (*SERPINE1*) and pyruvate dehydrogenase kinase 1 (*PDK1*). Subsequently, the key pathways tightly related to HIF-1 signaling were screened for those carrying a degree greater than 3, i.e., the focal adhesion and ErbB signaling pathways.

### Gene integration and hypoxia-associated gene validation

**Fig. 5:**
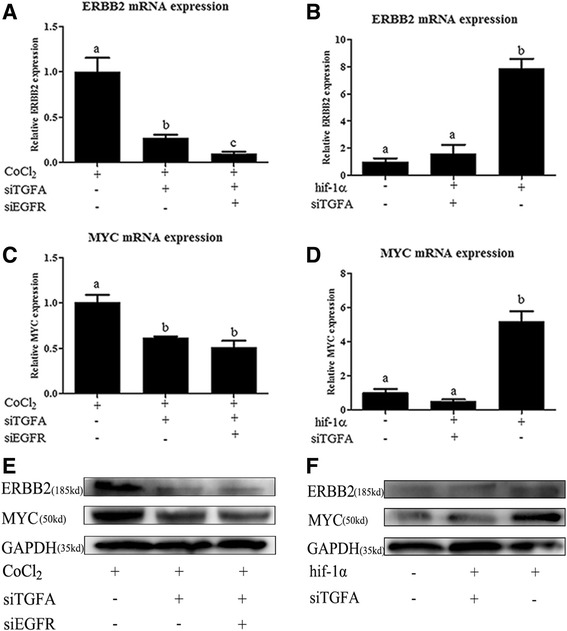
Validation of the selected genes in gene integration networks in Caov3 cells. Cells were exposed to hypoxia (treated with CoCl2 or transfected with hif-1α overexpression vector) followed by transiently transfected with shRNAs vector targeting TGFA, EGFR, or empty vector. Then total RNA and proteins were extracted for qRT-PCR and western blotting analysis. **a** and **b**, ErbB2 related mRNA expression; **c** and **d**, MYC related mRNA expression; E and F, ErbB2, MYC, GAPDH related protein expression, and GAPDH was used as a loading control. Values with different letters (**a**–**c**) differ significantly (*p* < 0.05)

To further understand the molecular pathways unique to ovarian cancer in hypoxia, it was essential to construct a gene network between pathways that were interlinked by one or more genes using simplified network diagrams. Thus, a linkage network between genes from the 11 pathways closely related to HIF-1 signaling was illustrated in the Additional file 3: Figure S3. Furthermore, to demonstrate the accuracy and reliability of the network constructed by bioinformatics analysis, a validation test was performed. Based on previous analysis, hub genes and node genes were both enriched in the ErbB signaling pathway. Thus, the ErbB signaling pathway was selected for verification. We investigated the link between HIF-1 signaling and ErbB signaling via TGFA and its receptor epidermal growth factor receptor (EGFR). Our study showed that exposing Caov3 cells to hypoxia (using cobalt chloride (CoCl_2_) or via transfection with a HIF-1α overexpression vector) induced the expression of *TGFA*, *EGFR* (data not shown) and the downstream genes such as *ErbB2* and *MYC* at mRNA and protein levels. The expression of these genes was decreased by interfering with TGFA and/or EGFR (Fig. 5).

### Cell proliferation assay

**Fig. 6:**
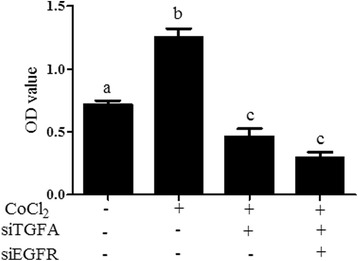
Validation of the proliferation of Caov3 cells by MTT assay. Values with different letters (**a**–**c**) differ significantly (*p* < 0.05)

Based on the literature, it is known that hypoxia can promote the invasion and adhesion of ovarian cancer, which ultimately affect the proliferation of cells. To further evaluate the importance of the network, the MTT assay was carried out in Caov3 cells to detect the multiplication capacities of the different treatment groups and verify the connection between HIF-1 signaling and ErbB signaling. It was shown that the proliferation of ovarian cancer cells was significantly promoted by treatment with CoCl_2_; however, this effect was significantly suppressed followed by transfection with shTGFA and/or shEGFR (Fig. 6).

## Discussion

Ovarian cancer accounts for approximately 4% of newly-diagnosed cancers in women. Despite the tireless efforts of researchers and advances in surgery and chemotherapy, ovarian cancer is still responsible for about 5% of female deaths caused by malignant neoplasms [41]. Therefore, searching for new drug targets and biomarkers that could facilitate the diagnosis and treatment of ovarian cancer is of great importance. Studies have shown that hypoxia plays an important role in the proliferation of ovarian cancer [42]. However, the exact mechanisms underlying how hypoxia regulates the growth of ovarian cancer cells is still not well understood. Here, we used bioinformatics methods to explore the regulatory network of gene expression profiling in ovarian cancer under hypoxic conditions.

In this study, a total of 931 overlapping DEGs were selected between cycling hypoxia and chronic hypoxia based on the datasets of GSE53012. A PPI network of these genes was constructed and hub genes (including *JUN*, *FOS*, *BIRC5*, *ESR1*, *MMP2*, *ErbB2*, *E2F7*, *MYC* and *VIM*) were identified by the STRING database and Cytoscape. Furthermore, pathway and functional enrichment analysis were performed followed by cluster analysis based on the KEGG database and the GO database to screen the key pathways and genes to provide insights into the physiological functions and progress of ovarian cancer under hypoxic conditions at the molecular level. The results indicate that the biological effects of hypoxia are mainly related to the proliferation, invasion, and adhesion of cells, such as regulation of cell proliferation, positive regulation of cell migration, focal adhesion and extracellular matrix binding. Moreover, the pathways closely related to the HIF-1 signaling pathway were selected with the cut off of the degree larger than 3, including focal adhesion, the ErbB signaling pathway, proteoglycans in cancer, the TNF signaling pathway, osteoclast differentiation, leukocyte transendothelial migration, pancreatic cancer, Chagas disease (American trypanosomiasis), central carbon metabolism in cancer, endometrial cancer, and prostate cancer. Interestingly, the regulatory pathways of hypoxia in other cancers were also present in ovarian cancer, indicating that a single signaling pathway might be involved in the formation of multiple cancers. In addition, the DEGs that only exist in cycling hypoxia were also explored through pathway analysis, including the Foxo signaling pathway, the MAPK signaling pathway and the cell cycle, which were mainly involved in the growth and development of cells and cell cycle regulation. Therefore, it was indicated that the periodicity of cell growth might be involved in the rhythmic regulation of hypoxia. It is worth noting that almost of all the hub genes and node genes were present in the ErbB signaling pathway, such as *TGFA*, *ErbB2*, and *MYC*. Accordingly, we propose the hypothesis that hypoxia promotes the proliferation of ovarian cancer cells mainly by the *HIF-1α-TGFA-EGFR-ErbB2-MYC* regulation axis and is dependent on the ErbB signaling pathway (Additional file 4: Figure S4).

The ErbB pathway involves a family of tyrosine kinases and contributes to resistance to radiation and chemotherapy in many tumor types, including ovarian cancer; its stimulation results in the proliferation of cells [43]. ErbB2, a member of the human epidermal growth factor receptor (HER/EGFR/ERBB) family, has been reported to be closely related to the occurrence and development of breast cancer [44, 45]. Moreover, a previous study indicated that the expression of *ErbB2* was positively correlated with the malignant potential of serous ovarian neoplasms [46]. However, there are also studies showing that serum VEGF levels might be used for diagnosis in ovarian cancer patients, while serum ErbB2 levels do not have a clinical significance in terms of ovarian cancer [47]. Interestingly, it has been reported that the level of VEGF in serum may depend on the expression of *ErbB2* in patients with ovarian cancer [48]. Therefore, the specific regulation of ErbB2 in ovarian cancer remains to be studied further. *MYC*, known as a regulatory gene, has been found to encode a multifunctional nuclear phosphoprotein which plays an important role in cell cycle progression, apoptosis, and cellular transformation [49]. The expression of *MYC* was increased by hypoxia in ovarian cancer cells, which may contributed to the observed resistance to platinum compounds. Moreover, ovarian cancer patients with high *MYC* mRNA levels tend to have lower disease-free (DFS) and the overall survival (OS) compared with their counterparts; thus, MYC may serve as a potential therapeutic target for ovarian cancers expressing high levels of this oncoprotein [50]. It has also been found that the disordered expression of *MYC* is common in human cancers and is closely related to the maintenance of aggressive of cancer stem cell populations. Additionally, HIF-1α expression has been significantly correlated with the expression of *MYC* and survivin in lung cancer [51]. EGFR, as an important receptor of TGFA, has attracted wide attentions from researchers. Previous studies have found that the frequency of oncogenic mutations in the *EGFR* gene is closely linked to the occurrence of non-small cell lung cancer (NSCLC). Moreover, EGFR has been proposed as a crucial molecular target for cancer therapy, promoting considerable research into the development of pharmacological inhibitors of EGFR [52–54]. Furthermore, Du et al. found that the expression of *EGFR* is closely correlated with progression-free survival (PFS) in post-operative patients with colorectal carcinoma, as patients with high *EGFR* expression were at a higher risk of tumor progression when comparing with their counterparts [55]. To our knowledge, however, there is no published study that has explored the effect of *EGFR* expression on ovarian cancer cell lines that have been exposed to hypoxia. Thus, EGFR may be a new therapeutic target for ovarian cancer, and may break through the bottleneck of current ovarian cancer treatment.

Admittedly, there were also some limitations to this study, since only the networks between genes and signaling pathways were explored in hypoxia while relevant transcription factor (TFs) and genes were not. Moreover, our results are mostly based on literature searches or bioinformatics predictions; thus, further validation is required and necessary. In addition, further in vivo studies are needed because of the growth mechanisms of cancer cells are so diverse that they can modify their migration mechanisms in response to different conditions in vitro.

## Conclusions

In conclusion, hub genes (such as *TGFA*, *EGFR*, *ErbB2*, and *MYC*) and key pathways (such as the ErbB signaling pathway) closely related to the proliferation of ovarian cancer cells in hypoxia were identified by bioinformatics analysis. Functional enrichment analysis revealed that these genes were mainly involved in the processes of cells proliferation, invasion, and adhesion. Additionally, the regulation of ErbB signaling by hypoxia was demonstrated by RT-PCR, WB, and MTT assays. These findings indicated that hypoxia regulated the growth of ovarian cancer cells mainly through regulation of the *HIF-1α-TGFA-EGFR-ErbB2-MYC* axis, which might provide a new drug target and biomarker and lead to improved insights on diagnosis and treatment of ovarian cancer.

## Additional files

Additional file 1: **Figure S1.** The KEGG pathway analysis of the most significant module. The number of DEGs in each signaling pathway was presented in different gradually changing color from red to blue. (PNG 563 kb)

Additional file 2: **Figure S2.** Integration of all signaling pathways. Gene products were visualized as blue ellipses. The HIF-1 signaling pathway was marked by a purple ellipse, and signaling pathways marked as orange ellipses represented pathways closely related to the HIF-1 signaling pathway, and other signaling pathways were marked by red ellipses. (PNG 952 kb)

Additional file 3: **Figure S3.** Integration of genes of signaling pathways. Genes in the network were indicated as blue ellipses and pathways as red ellipses. In addition, HIF-1 signaling pathway, ErbB2, TGFA, MYC were tagged with purple ellipses as they would be validated in the following tests. (PNG 900 kb)

Additional file 4: **Figure S4.** ErbB signaling pathway. This map of ErbB signaling pathway was obtained based on KEGG. The red boxes represented overlapping DEGs between cycling hypoxia and chronic hypoxia which were verified by previous analysis. (PNG 799 kb)

## Abbreviations

BCL6: B-cell CLL/lymphoma 6
BP: Biological process
CC: Cellular component
CCNB1: Cyclin B1
CCNB2: Cyclin B2
CCNE1: Cyclin E1
CDC20: Cell division cycle 20
CoCl2: Cobalt chloride
CYP3A5: Cytochrome P450 family 3 subfamily A member 5
DAVID: Database for annotation, Visualization and integrated discovery
DEGs: Differentially expressed genes
EOC: Epithelial ovarian cancer
FDG: Fluorodeoxyglucose
FOXO3: Forkhead box O3
GEO: Gene expression omnibus
GO: Gene ontology
hif-1: Hypoxia inducible factor-1
IL-8: Interleukin-8
KEGG: Kyoto encyclopedia of genes and genomes
MF: Molecular function
NCBI: National center of biotechnology information
PPI: Protein-protein interaction
RPS6KA6: Ribosomal protein S6 kinase A6
STRING: The Search Tool for the retrieval of interacting genes database
VEGF: Vascular-endothelial growth factor
VEGFA: Vascular endothelial growth factor A
