# Supplementary figures and images for: Investigation of hypoxia networks in ovarian cancer via bioinformatics analysis
Source: J Ovarian Res. 2018 Feb 26;11:16. doi: 10.1186/s13048-018-0388-x (PMC5828062; doi:10.1186/s13048-018-0388-x)

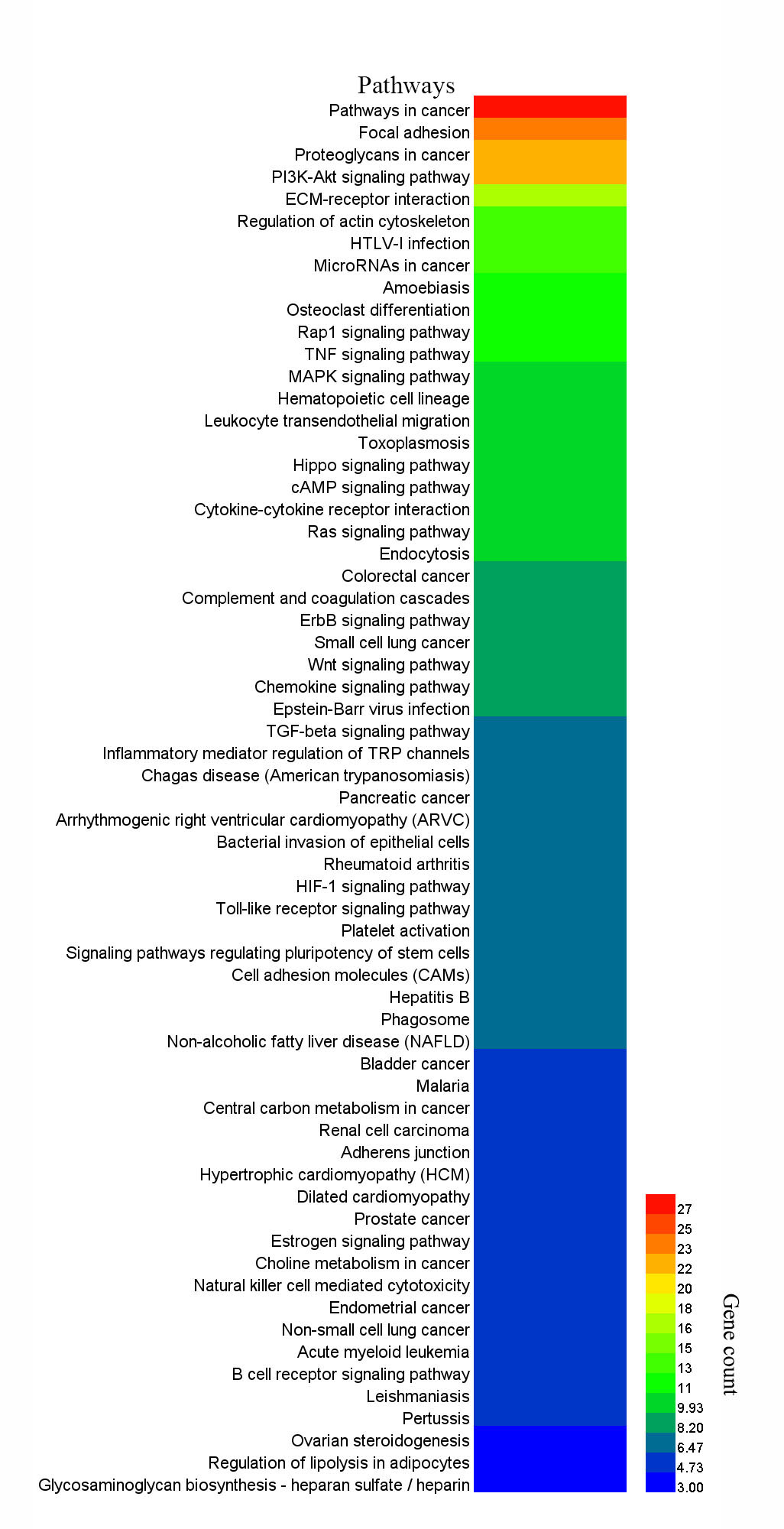

Supplement: Supplementary file 1 — Figure S1. The KEGG pathway analysis of the most significant module. The number of DEGs in each signaling pathway was presented in different gradually changing color from red to blue. (PNG 563 kb) [file 13048_2018_388_MOESM1_ESM.png]

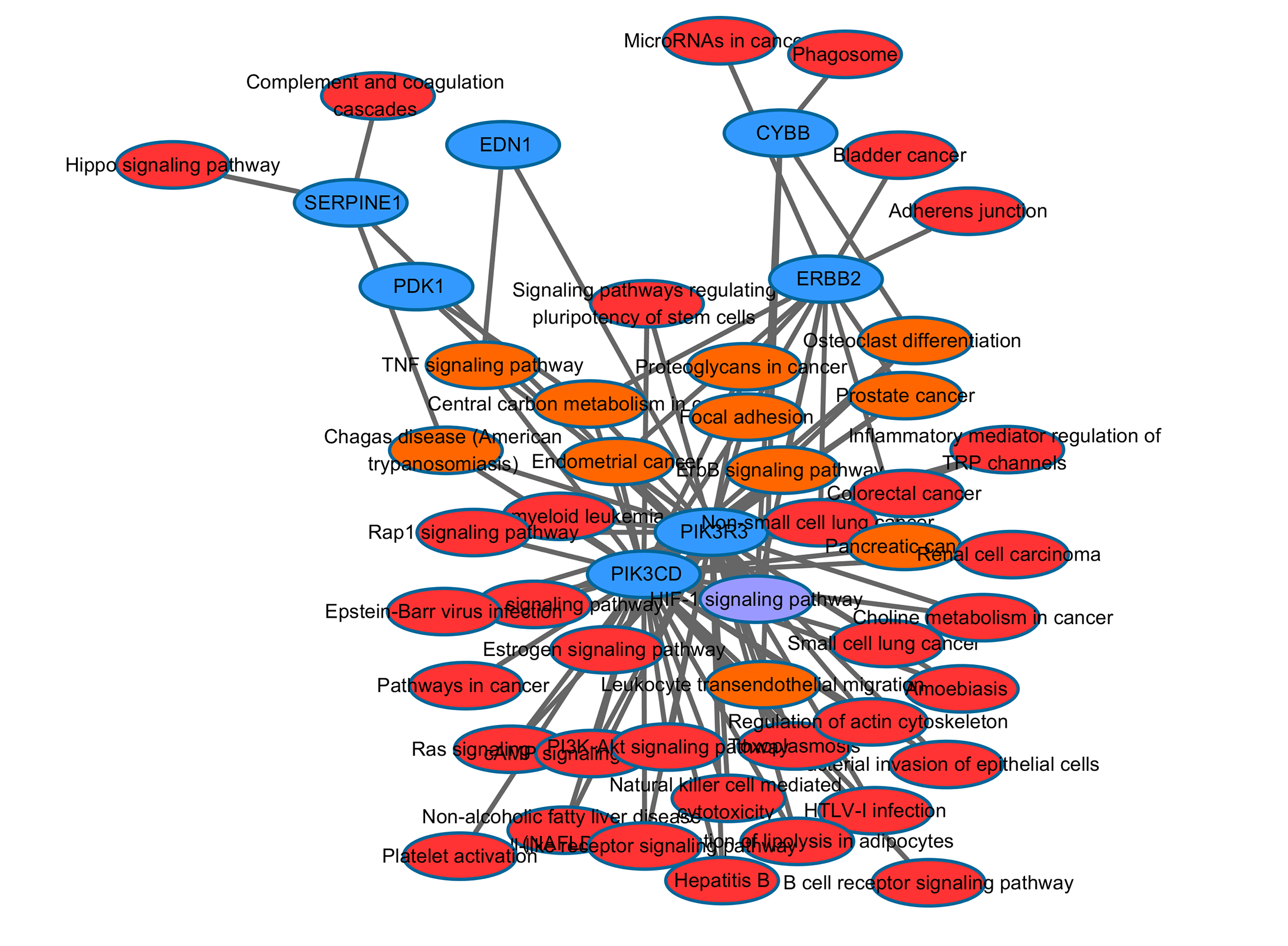

Supplement: Supplementary file 2 — Figure S2. Integration of all signaling pathways. Gene products were visualized as blue ellipses. The HIF-1 signaling pathway was marked by a purple ellipse, and signaling pathways marked as orange ellipses represented pathways closely related to the HIF-1 signaling pathway, and other signaling pathways were marked by red ellipses. (PNG 952 kb) [file 13048_2018_388_MOESM2_ESM.png]

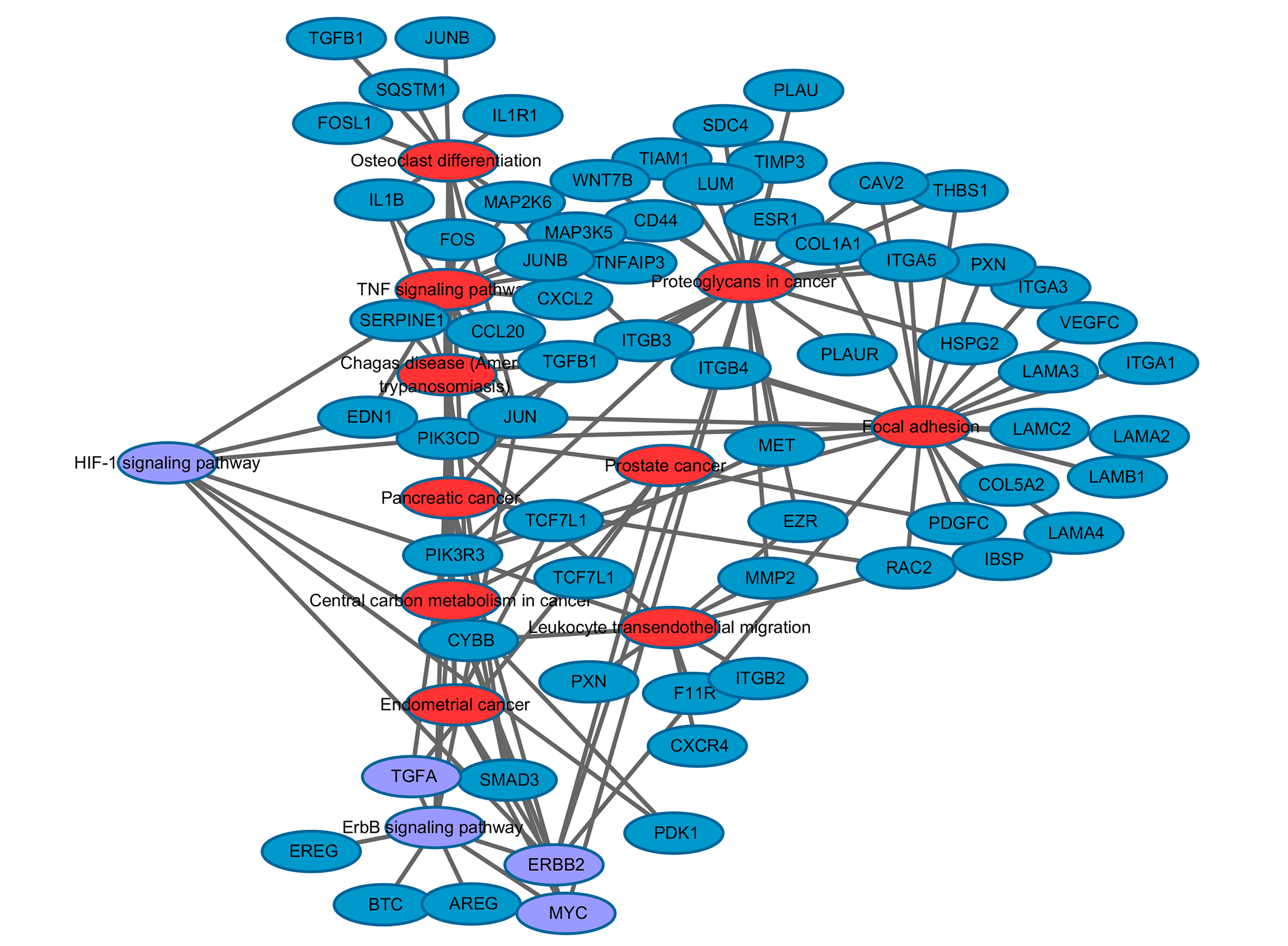

Supplement: Supplementary file 3 — Figure S3. Integration of genes of signaling pathways. Genes in the network were indicated as blue ellipses and pathways as red ellipses. In addition, HIF-1 signaling pathway, ErbB2, TGFA, MYC were tagged with purple ellipses as they would be validated in the following tests. (PNG 900 kb) [file 13048_2018_388_MOESM3_ESM.png]

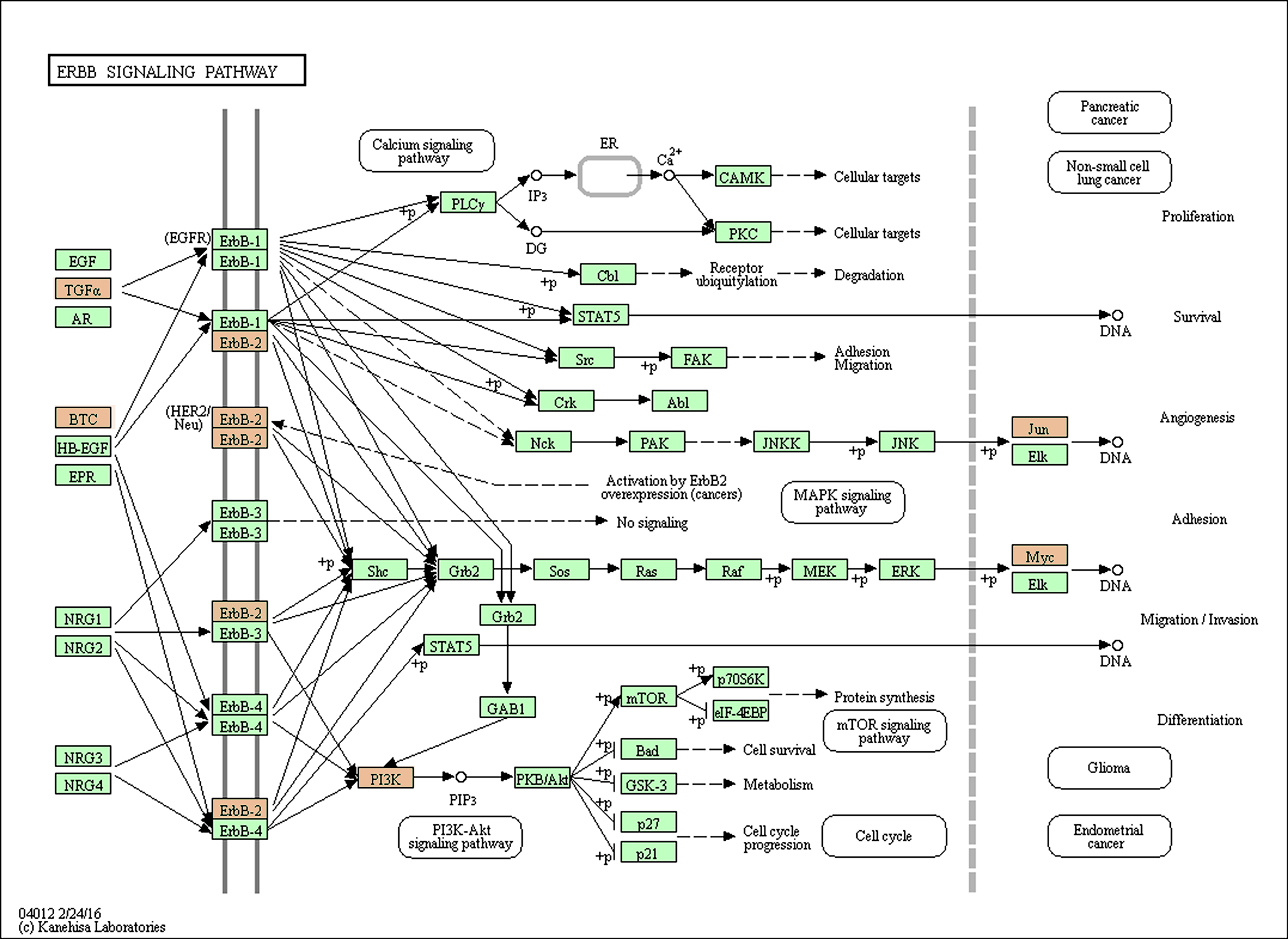

Supplement: Supplementary file 4 — Figure S4. ErbB signaling pathway. This map of ErbB signaling pathway was obtained based on KEGG. The red boxes represented overlapping DEGs between cycling hypoxia and chronic hypoxia which were verified by previous analysis. (PNG 799 kb) [file 13048_2018_388_MOESM4_ESM.png]
